# Protective Effect of Vanillic Acid against Hyperinsulinemia, Hyperglycemia and Hyperlipidemia via Alleviating Hepatic Insulin Resistance and Inflammation in High-Fat Diet (HFD)-Fed Rats

**DOI:** 10.3390/nu7125514

**Published:** 2015-12-02

**Authors:** Wen-Chang Chang, James Swi-Bea Wu, Chen-Wen Chen, Po-Ling Kuo, Hsu-Min Chien, Yuh-Tai Wang, Szu-Chuan Shen

**Affiliations:** 1 Graduate Institute of Food Science and Technology, National Taiwan University, P.O. Box 23-14, Taipei 10672, Taiwan; d99641001@ntu.edu.tw (W.-C.C.); jsbwu@ntu.edu.tw (J.S.-B.W.); jenwen0813@hotmail.com (C.-W.C.); 2 Department of Human Development and Family Studies, National Taiwan Normal University, No. 162, Sec. 1, Heping East Road, Taipei 10610, Taiwan; maplebling@gmail.com; 3 Department of Nursing, Taipei City Hospital, Renai Branch, No. 10, Sec. 4, Renai Road, Taipei 10629, Taiwan; B1465@tpech.gov.tw; 4 Life Science Center, Hsing Wu Institute of Technology, No. 101, Sec. 1, Fen-Liao Road, Lin-Kou District, New Taipei City 244, Taiwan; yuhtai@yahoo.com

**Keywords:** vanillic acid, insulin resistance, hyperinsulinemia, hyperglycemia, hyperlipidemia

## Abstract

Excess free fatty acid accumulation from abnormal lipid metabolism results in the insulin resistance in peripheral cells, subsequently causing hyperinsulinemia, hyperglycemia and/or hyperlipidemia in diabetes mellitus (DM) patients. Herein, we investigated the effect of phenolic acids on glucose uptake in an insulin-resistant cell-culture model and on hepatic insulin resistance and inflammation in rats fed a high-fat diet (HFD). The results show that vanillic acid (VA) demonstrated the highest glucose uptake ability among all tested phenolic acids in insulin-resistant FL83B mouse hepatocytes. Furthermore, rats fed HFD for 16 weeks were orally administered with VA daily (30 mg/kg body weight) at weeks 13–16. The results show that levels of serum insulin, glucose, triglyceride, and free fatty acid were significantly decreased in VA-treated HFD rats (*p* < 0.05), indicating the protective effects of VA against hyperinsulinemia, hyperglycemia and hyperlipidemia in HFD rats. Moreover, VA significantly reduced values of area under the curve for glucose (AUC_glucose_) in oral glucose tolerance test and homeostasis model assessment-insulin resistance (HOMA-IR) index, suggesting the improving effect on glucose tolerance and insulin resistance in HFD rats. The Western blot analysis revealed that VA significantly up-regulated expression of hepatic insulin-signaling and lipid metabolism-related protein, including insulin receptor, phosphatidylinositol-3 kinase, glucose transporter 2, and phosphorylated acetyl CoA carboxylase in HFD rats. VA also significantly down-regulated hepatic inflammation-related proteins, including cyclooxygenase-2 and monocyte chemoattractant protein-1 expressions in HFD rats. These results indicate that VA might ameliorate insulin resistance via improving hepatic insulin signaling and alleviating inflammation pathways in HFD rats. These findings also suggest the potential of VA in preventing the progression of DM.

## 1. Introduction

As advanced medical technology and improved living standards extend the expectancy of human life, chronic diseases have become a major threat to the health of people. Diabetes mellitus (DM) is one of the fastest-growing chronic diseases. The World Health Organization (WHO) reported that there were more than 347 million people suffering from DM worldwide, and predicted the patient number would double (694 million) by 2030 [1]. The process of DM development involves an initial prediabetes state, and develops into DM if the condition is not appropriately controlled. Therefore, effectively maintaining blood glucose homeostasis has become a crucial issue in DM prevention.

After consuming high-calorie foods, the body is in a hyperglycemic state with an increased insulin secretion, reducing serum glucose to maintain a steady level of serum glucose. Long-term excess intake of high-calorie or high-fat diet (HFD) resulted in increasing blood glucose level and free fatty acid content and induced metabolic related diseases, such as obesity, dyslipidemia, type 2 diabetes mellitus (T2DM), and fatty liver disease [2,3,4]. The increased blood glucose level caused islet cells to continuously secrete insulin, while the increased free fatty acid content caused an increased lipid synthesis, leading to the accumulation of diacylglycerol in the liver and activation of protein kinase Cε (PKCε). Activated PKCε inhibits the insulin signaling, consequently resulting in hepatic insulin resistance [4]. HFD also tends to be obesity associated. Previous studies indicated that insulin resistance that occurred via obesity is correlated with internal chronic inflammation, which is among the major causes of insulin resistance [5,6,7]. Increased release of free fatty acids from adipocytes into blood was reported to activate protein kinases, e.g., protein kinase C, which subsequently affected the expression of inhibitor of kappa β kinase, c-Jun N-terminal kinases (JNK), and p38 mitogen-activated protein kinases, stimulating the release of inflammatory factors such as tumor necrosis factor-alpha (TNF-α) and interleukin-6 (IL-6), and causing internal inflammatory responses [8].

Phenolic acids are phytochemicals abundant in various vegetables and fruits. Hydroxybenzoic acid derivatives and hydroxycinnamic acid derivatives are two major categories of phenolic acid [9]. Hydroxycinnamic acid derivative *p*-methoxycinnamic acid promoted glycolysis, reduced gluconeogenesis in the liver of diabetic rats, and increased the secretion of insulin to reduce hyperglycemia in streptozotocin (STZ)-induced diabetic rats [10]. Ferulic acid improved the serum glucose levels and counteracted lipid peroxidation in STZ-induced diabetic mice and KK-Ay spontaneous diabetic mice [11]. Caffeic acid was reported to increase the utilization of glucose by the liver and adipocytes to reduce serum glucose levels in type 2 diabetic mice [12]. Various phenolic acids were found to alleviate the DM and its associated syndromes [9,10,11,12]. However, the study of phenolic acids against HFD-induced hyperinsulinemia, hyperglycemia and hyperlipidemia is limited. The aim of the present study is to assess the glucose uptake-enhancing effect *in vitro* and investigate the hypoinsulinemic, hypoglycemic and hypolipidemic effect *in vivo* of phenolic acids. The mechanism of the selected phenolic acid on attenuating insulin resistance in HFD rats is also elucidated.

## 2. Materials and Methods

### 2.1. Chemicals

Bovine serum albumin (BSA), caffeic acid, chlorogenic acid, cinnamic acid, d-(+)-glucose, dimethyl sulfoxide (DMSO), disodium hydrogen phosphate (Na_2_HPO_4_), ferulic acid, 4-(2-hydroxyethyl)-1-piperazineethanesulfonic acid (HEPES), insulin, pioglitazone hydrochloride (Pio), potassium chloride (KCl), potassium dihydrogen phosphate (KH_2_PO_4_), protocatechuic acid, sinapic acid, sodium chloride (NaCl), sodium phosphate dibasic (Na_2_HPO_4_), syringic acid, vanillic acid (VA), recombinant mouse tumor necrosis factor (TNF)-α, sulfuric acid (H_2_SO_4_), Triton X-100, TEMED (*N*,*N*,*N*,*N*′-Tetramethyl-eyhylenediamine), 3-(4,5-dimethylthiazol-2-yl)-2,5-diphenyl-tetrazolium bromide (MTT reagent), and F12 Ham Kaighn’s modification (F12K) medium were purchased from Sigma-Aldrich Co. (St. Louis, MO, USA). Fetal bovine serum (FBS) was obtained from Gemini Bio-Products (Woodland, CA, USA). The fluorescent dye 2-(N-(7-nitrobenz-2-oxa-1,3-diazol-4-yl)amino)-2-deoxyglucose (2-NBDG) was purchased from Invitrogen (Camarillo, CA, USA). Bio-Rad protein assay dye reagent was obtained from Bio-Rad Laoboratories (Richmond, VA, USA). All of the chemicals used in this study were of analytical grade.

### 2.2. Cell Culture

Experiments were performed on a hepatocyte cell line (FL83B) deriving from a fetal mouse (15–17 days). The FL83B cells were incubated in F12K medium containing 10% fetal bovine serum and 1% penicillin and streptomycin (Invitrogen Corporation, Camarillo, CA, USA) in 10 cm Petri dishes at 37 °C and 5% carbon dioxide. Experiments were performed when cells were 80%–90% confluent.

### 2.3. Tumor Necrosis Factor-Alpha (TNF-*α*) Induction of Insulin Resistance

The induction of insulin resistance in hepatocytes referred to the method reported by Chang and Shen with minor modifications [13]. FL83B cells were seeded in 10 cm dishes and incubated at 37 °C for 48 h to 80% confluence. Serum-free F12K medium containing 20 ng/mL recombinant mouse TNF-α was then added and incubated for 5 h to induce insulin resistance.

### 2.4. Uptake of Fluorescent 2-(N-(7-Nitrobenz-2-oxa-1,3-Diazol-4-yl)Amino)-2-Deoxyglucose in FL83B Mouse Hepatocytes

The FL83B cells were detached with trypsin and suspended in 1200 μL of Krebs-Ringer bicarbonate buffer containing 1 μM insulin. Aliquots of the cell suspension (172 μL) were transferred to Eppendorf tubes and co-incubated with 20 μL of 6.25 ng/mL VA and 8 μL of the fluorescent dye 2-NBDG (to a final concentration of 200 μM) in a water bath at 37 °C for 1 h in the dark. The reaction was stopped on ice. The cell suspension was centrifuged at 3000× *g* (4 °C) for 5 min to remove the supernatant. The pellet was washed with phosphate-buffered saline (PBS) and centrifuged 3 times before being suspended in 1 mL of PBS. The fluorescence intensity of the cell suspension was evaluated using flow cytometry (FACScan, Becton Dickinson, Bellport, NY, USA) at an excitation wavelength of 488 nm and an emission wavelength of 542 nm. Fluorescence intensity reflected the cellular uptake of 2-NBDG.

Amelioration rate (%) = ((fluorescence intensity of phenolic acid-treated group) − (fluorescence intensity of TNF-α-treated group))/(fluorescence intensity of TNF-α-treated group) × 100 (%).
(1)

### 2.5. Animals and Diets

Male Sprague-Dawley (SD) rats (5 weeks old) were obtained from the National Laboratory Animal Center, Taipei, Taiwan. The rats were maintained in standard laboratory conditions (22 ± 1 °C and a 12 h light/12 h dark cycle) with free access to food and water. Rats were fed a normal diet for 1 week and had a body weight of approximately 250 g. The rats were divided into 4 groups, with each group containing 6 rats. One group was fed a normal diet for 16 weeks (Control group). A second group was fed an HFD (60% calories from fat) throughout the experimental period (HFD group). A third group was provided an HFD for 16 weeks and daily administered Pio (30 mg/kg body weight) on a daily basis during weeks 13–16 (HFD + Pio group). A final group was provided an HFD for 16 weeks, and orally administered VA (30 mg/kg body weight) on a daily basis during weeks 13–16 (HFD + VA group). The rats were sacrificed at the end of the experiment before the blood samples were collected and the biochemical analysis conducted. The organs such as liver, kidney, perirenal and epididymal adipose tissues were isolated from animals and weighed. The liver was stored at −80 °C for the free fatty acid assay and Western blot analysis.

### 2.6. Blood Sample Preparation

Blood samples were collected and allowed to clot for 30 min at room temperature and then centrifuged at 3000× *g* for 20 min to obtain the serum, which was stored at −80 °C before use.

### 2.7. Biochemical Measurements

Enzyme-linked immunosorbent assay kits for rat insulin, total bilirubin, blood urea nitrogen, creatinine, total cholesterol, triglyceride, free fatty acid, and leptin were purchased from Randox Laboratories (Crumlin Co., Antrim, UK). Biochemical analyses were performed according to the manufacturer’s protocols.

### 2.8. Oral Glucose Tolerance Test (OGTT)

The OGTT was performed on rats in all groups after an overnight fast at week 16. All animals were orally administered 1.5 g of glucose/kg body weight. Blood was sampled from the tail vessels of conscious animals before (t = 0) and 30, 60, 90, and 120 min after glucose administration. The samples were allowed to clot for 30 min and then centrifuged (4 °C, 3000× *g*, 20 min) to obtain the serum. Glucose concentration was determined using a glucose enzymatic kit (Crumlin Co.). The obtained glucose concentration values were plotted against time to provide a curve showing the changes in glucose levels with time, expressed as an integrated area under the curve for glucose (AUC_glucose_).

### 2.9. Homeostasis Model Assessment of Insulin Resistance (HOMA-IR) Index [14]

The HOMA-IR index was calculated using the following equation:

HOMA-IR index = fasting serum insulin (mU/L) × fasting glucose (mmol/L)/22.5.
(2)

### 2.10. Western Blot Analysis

Aliquots of supernatants, each containing 50 μg protein, were used to evaluate the expression of insulin receptor (IR), phosphatidylinositol-3 kinase (PI3K), glucose transporter 2 (GLUT-2), cyclooxygenase-2 (COX-2), monocyte chemoattractant protein-1 (MCP-1), and acetyl CoA carboxylase (ACC), phospho-acetyl CoA carboxylase (pACC). The samples were subjected to 10% sodium dodecyl sulfate polyacrylamide gel electrophoresis, and the proteins were electrotransferred to a polyvinylidene difluoride membrane. The membrane was incubated with block buffer (PBS containing 0.05% Tween-20 and 5% *w*/*v* nonfat dry milk) for 1 h, washed with PBS containing 0.05% Tween-20 (PBST) 3 times, and then probed with 1:2000 diluted solutions of anti-IR, anti-PI3K, anti-GLUT-2, anti-COX-2, anti-MCP-1, and anti-ACC, anti-pACC, antibodies (Gene Tex, Irvine, CA, USA) overnight at 4 °C. The intensity of the blot probed with a 1:4000 diluted solution of mouse monoclonal antibody to bind actin (Gene Tex) was used as a control to ensure that a constant amount of protein was loaded into each lane of the gel. The membrane was washed 3 times (5 min each time) in PBST, shaken in a solution of horseradish peroxidase-linked anti-mouse IgG or anti-rabbit IgG secondary antibody, washed 3 more times (5 min each time) in PBST, and then exposed to enhanced chemiluminescence reagent (Millipore) according to the manufacturer’s instructions. The films were scanned and analyzed using a UVP Biospectrum image system (Level, Cambridge, UK).

### 2.11. Statistical Analysis

Results presented as mean ± standard deviation (SD) were analyzed using one-way ANOVA and Duncan’s new multiple range tests. All comparisons were made relative to controls, and *p* < 0.05 was considered significant.

## 3. Results and Discussion

### 3.1. Effect of Phenolic Acids on Cell Viability and Glucose Uptake Ability in Insulin-Resistant FL83B Mouse Hepatocytes

The *in vitro* MTT assay is typically used to evaluate the cellular growth inhibition and assess toxicity of drugs or chemicals [15]. Phenolic acids are known to possess antiviral, antioxidative, anticancer, and antihyperglycemic bioactivities [16,17,18]. However, the effect of phenolic acid on bioactivity and the cytotoxicity in FL83B mouse hepatocytes have not been systematically investigated. Table 1 shows the growth inhibitory of concentration effect of eight naturally occurred phenolic acids on FL83B cells. The results from the cell viability test reveal that the all tested phenolic acids at the concentration of 12.5 μM show above 80%, indicating non-toxicity to FL83B mouse hepatocytes. Therefore, this concentration (12.5 μM) was used to evaluate the glucose uptake-enhancing effect of phenolic acids in insulin resistance cell model.

**Table 1:** Effects of various concentrations of phenolic acids on cell viability and amelioration rate of glucose uptake in insulin-resistant FL83B cells.

	Concentrations	Cell Viability (%) ^a^				Amelioration Rate (%) ^b^ (12.5 μM Phenolic Acid)
Phenolic Acids		12.5 μM	25 μM	50 μM	100 μM	
Caffeic acid		102.8 ± 15.8	99.4 ± 16.8	82.0 ± 14.3	74.3 ± 8.6	−6.60
Cinnamic acid		86.2 ± 2.1	88.7 ± 2.5	88.6 ± 6.7	89.6 ± 0.1	−11.30
Ferulic acid		92.7 ± 7.3	88.9 ± 9.1	86.7 ± 6.7	77.4 ± 28.2	−22.01
Protocatechuic acid		91.7 ± 10.4	91.8 ± 9.0	75.4 ± 8.1	71.8 ± 9.0	−12.90
Rosmarinic acid		104.3 ± 17.6	97.2 ± 13.1	102.5 ± 16.5	90.7 ± 22.4	−10.28
Sinapic acid		97.2 ± 2.9	102.1 ± 10.1	102.0 ± 10.5	80.0 ± 9.2	−15.37
Syringic acid		90.9 ± 2.2	79.8 ± 17.4	70.2 ± 13.1	64.6 ± 14.5	8.36
Vanillic acid		114.2 ± 26.3	114.8 ± 18.5	109.8 ± 25.5	116.7 ± 17.6	13.66

^a^ Cell viability (%) = (A_570_ of sample group)/(A_570_ of control group) × 100 (%); ^b^ Amelioration rate (%) = ((fluorescence intensity of phenolic acid-treated group) − (fluorescence intensity of TNF-α-treated group))/(fluorescence intensity of TNF-α-treated group) × 100 (%). Data are expressed as percentage relative to control value (100%) or mean ± SD (*n* = 3).

TNF-α is a cytokine found to interfere with the transmission of insulin signaling and the abilities of liver cells, myofibroblasts, and adipocytes to absorb and metabolize glucose [19,20,21]. Previously, the 2-NBDG, a modified d-glucose fluorescent derivative, was used to assess the viability of yeast and *Escherichia coli*, and the ability of glucose uptake in FL83B cells treated with TNF-α to induce insulin resistance [13,22,23]. In the present study, fluorescent 2-NBDG was used to evaluate the effect of phenolic acids on glucose uptake ability in insulin-resistant FL83B mouse hepatocytes. As shown in Table 1, VA at concentration of 12.5 μM showed the highest potential for reducing insulin resistance (amelioration rate, 13.66%) among tested phenolic acids. VA was thus used in the subsequent animal experiments.

### 3.2. Effect of Vanillic Acid on Glucose Tolerance, Serum Insulin and Insulin Resistance Index in High-Fat Diet (HFD)-Fed Rats

**Figure 1:**
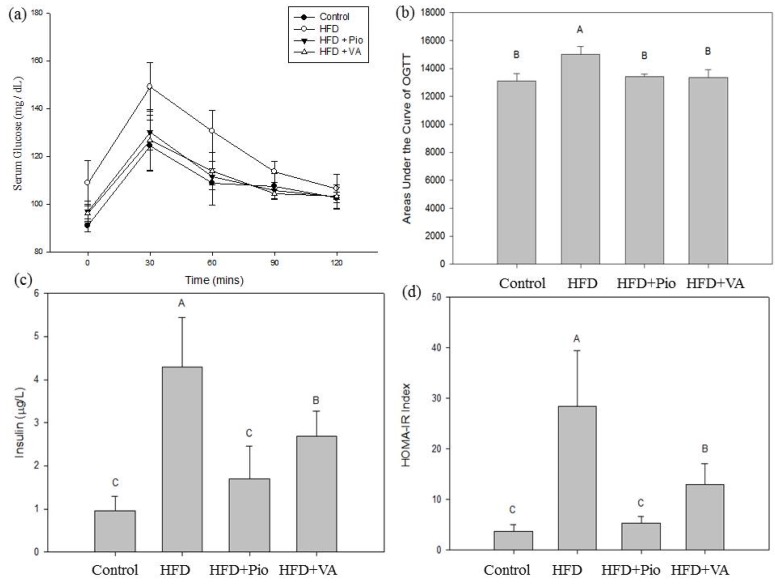
(**a**) Oral glucose tolerance test (OGTT); (**b**) area under the curve for glucose (AUC_glucose_) of OGTT; (**c**) fasting serum insulin, and (**d**) homeostasis model assessment of insulin resistance (HOMA-IR) in rats fed high-fat diet for 16 weeks and orally administered with vanillic acid during the last 4 weeks. Control: normal diet; HFD: high-fat diet (60 kcal % fat); HFD + Pio: HFD (60 kcal % fat) + pioglitazone (30 mg/kg body weight); HFD + VA: HFD (60 kcal % fat) + vanillic acid (30 mg/kg body weight); (A–C) indicate statistically significant differences *p* < 0.05. Data are presented as mean ± SD (6 rats in each group).

HFD is associated with insulin resistance and reduced insulin secretion by β-cells in the pancreas, leading to abnormal glucose tolerance in animal [24,25,26,27]. Figure 1a shows the results of OGTT in rats fed HFD for 16 weeks and orally administered with VA daily during the last 4 weeks. The serum glucose levels of rats in the HFD group increased significantly after 30 min, and then decreased slowly. HFD rats treated with Pio or VA exhibited similar changes in serum glucose levels. The AUC_glucose_ for the OGTT, indicating the degree of glucose tolerance in the rats, remained at high levels, thereby indicating low glucose tolerance in the present study. The HFD rats exhibited significantly (*p* < 0.05) lower glucose tolerance than the control rats and VA-treated HFD rats (Figure 1b). In other words, VA can ameliorate the HFD-induced glucose intolerance.

Hyperinsulinemia is likely a marker of insulin resistance, rather than a major, direct contributor to the process [28]. Under hyperglycemic conditions, the pancreas compensates for the decreased insulin response by increasing the insulin secretion; however, the result is hyperinsulinemia to maintain the stable plasma glucose. This process will continue until the reserve capacity is surpassed by metabolic demands and insulin secretion is no longer sufficient; then, blood glucose concentration rises and glucose intolerance and T2DM develop [29,30,31]. HFD is also reported to be associated with the hyperinsulinemia of rats [32]. In this study, the fasting serum insulin concentration of rats in the HFD group (4.30 ± 1.14 μg/L) was 347.9% higher than that in the control (normal diet) group (0.96 ± 0.33 μg/L) (Figure 1c), indicating the hyperinsulinemia in the HFD rats. However, the serum insulin level of VA-treated HFD rats (2.13 ± 0.64 μg/L) was significantly lower than that in HFD-fed rats, indicating the hypoinsulinemic ability of VA. The rats fed HFD over the long term will develop diabetic symptoms such as insulin resistance [33]. Matthews *et al.* proposed methods using the fasting serum insulin and glucose levels during leisure time to calculate the HOMA-IR index, which is considered a sensitive indicator to assess the degree of insulin resistance [14]. A high HOMA-IR value represents high insulin resistance [14,34]. The advantages of the HOMA-IR are ease of calculation of fasting serum insulin and glucose levels, and potential for extensive use in epidemiological research. As shown in Figure 1d, the HOMA-IR index of VA-treated HFD rats was significantly lower than that of HFD rats, indicating VA may ameliorate the insulin resistance in HFD rats. VA derivative was previously reported to inhibit protein-tyrosine phosphatase 1B (PTP1B) activity, reduce the interference on insulin-signaling proteins, and lead to the alleviation of insulin resistance in T2DM patients [35].

### 3.3. Effect of Vanillic Acid on Energy Intake, Body Weight, and Selected Organ Weight in HFD-Fed Rats

No significant difference (*p* > 0.05) in the energy intakes among tested groups was observed, indicating that orally administered VA exerts no significant effect on energy intake in HFD rats (Table 2). There was no significant difference in the kidney weights of each group (Table 2). However, after 4 weeks of treatment, the average body, liver and adipose tissue weights of HFD rats were significantly higher than those of Pio and VA-treated HFD rats (*p* < 0.05). HFD was reported to increase serum triglyceride and non-esterified free fatty acid (NEFA) content, thereby resulting in increased lipogenesis in rats [36]. Pio was reported to restrict the increase of adipose tissue and body weight in obese women [37]. Thus, VA was postulated to possess the similar effect as Pio on suppressing accumulation of body fat in HFD-fed rats via the inhibition of lipid synthesis or lipogenesis.

**Table 2:** The body weight, selected tissue weight, and energy intake in rats fed a high-fat diet for 16 weeks and orally administered with vanillic acid during the last 4 weeks.

Items/Groups	Control	HFD	HFD + Pio	HFD + VA
Body weight (g)	627.1 ± 28.7 ^C^	760.7 ± 63.1 ^A^	686.0 ± 30.4 ^B^	676.9 ± 70.2 ^B^
Diet intake (kcal/rat/day)	104.45 ± 4.43 ^A^	110.04 ± 11.33 ^A^	101.63 ± 5.77 ^A^	104.97 ± 9.91 ^A^
Liver weight (g)	14.6 ± 0.7 ^B^	18.5 ± 2.2 ^A^	13.9 ± 1.4 ^B^	13.9 ± 1.6 ^B^
Kidney weight (g)	1.1 ± 0.4 ^A^	1.4 ± 0.5 ^A^	1.7 ± 0.2 ^A^	0.9 ± 0.6 ^A^
Adipose weight (g)	17.7 ± 4.6 ^C^	56.1 ± 12.7 ^A^	36.7 ± 6.4 ^B^	35.3 ± 12.7 ^B^

Control: normal diet; HFD: high-fat diet (60 kcal % fat); HFD+Pio: HFD (60 kcal % fat) + pioglitazone (30 mg/kg body weight); HFD + VA: HFD (60 kcal % fat) + vanillic acid (30 mg/kg body weight). Adipose weight: total weight of perirenal and epididymal adipose tissues. (^A^^–C^) indicate statistically significant differences *p* < 0.05. Data are presented as mean ± SD (*n* = 6/group). Adipose weight includes epididymal fat pad and abdominal adipose tissue weight.

### 3.4. Effect of Vanillic Acid on Serum Biochemical Parameters in HFD-Fed Rats

Long term consumption HFD resulting in the increment of adipose tissues. The fatty acids from adipocytes are subsequently released into the blood then transported to periphery tissues such as liver and muscle. The process inhibits the utilization of glycogen and glucose and induces insulin resistance in peripheral tissues. HFD was reported to induce dyslipidemia, resulting in elevated serum glucose, triglyceride, NEFA levels and reduced high-density lipoprotein levels in rats [33,38]. After feeding rats for 16 weeks, the fasting serum glucose of the HFD group was 18.1% higher than that of control group (Table 3). The significant difference (*p* < 0.05) between these two groups was consistent to the previous study and revealed that hyperglycemia was induced in HFD-fed rats in the present study [38]. Furthermore, the fasting serum glucose level in VA-treated rats (94.5 ± 1.9 mg/L) was significantly lower than that in the HFD rats (*p* < 0.05) (Table 3), indicating the hypoglycemic ability of VA.

Moreover, the fasting serum triglyceride and free fatty acid concentration of rats in the HFD group are higher than those in the control group by 20.8% and 27.2%, respectively (Table 3), indicating the hyperlipidemia in the HFD rats. However, the fasting serum triglyceride and free fatty acid were significantly reduced in HFD rats supplemented with VA (71.63 ± 13.42 μg/L, 0.76 ± 0.14 mmol/L) compared to the HFD rats (*p* < 0.05), suggesting the hypolipidemic ability of VA.

Leptin is a hormone secreted by adipose tissues and regulates utilization of lipid in human body. The serum leptin concentration of in the HFD rats was significantly (*p* < 0.05) higher than that in control group (Table 3). Comparatively, the serum leptin level was significantly reduced (*p* < 0.05) in HFD rats after 4-weeks VA treatment (Table 3). Previous studies reported a positive correlation between serum leptin concentration and body fat content, and suggested that people with obesity or T2DM have higher serum leptin levels than healthy people do [39,40]. Studies have also indicated that leptin reduces body weight, body fat, energy intake, serum glucose levels, and insulin concentration in mice [41,42]. However, the mechanism underlying such effects was still unidentified. We speculate that HFD promotes leptin production and is involved in the accumulation of hepatic NEFA and the induction of hepatic insulin resistance in rats subsequently.

**Table 3:** The fasting serum parameters in rats fed high-fat diet for 16 weeks and orally administered with vanillic acid during the last 4 weeks.

Items/Groups	Control	HFD	HFD + Pio	HFD + VA
Glucose (mg/dL)	90.8 ± 1.7 ^B^	107.2 ± 5.5 ^A^	97.0 ± 3.7 ^B^	94.5 ± 1.9 ^B^
Triglyceride (mg/dL)	74.13 ± 18.20 ^B^	89.50 ± 11.70 ^A^	61.50 ± 9.80 ^B^	71.63 ± 13.42 ^B^
Free fatty acid (mmol/L)	0.92 ± 0.09 ^B^	1.17 ± 0.26 ^A^	0.69 ± 0.16 ^C^	0.76 ± 0.14 ^B,C^
Total cholesterol (mg/dL)	62.38 ± 9.71 ^A^	51.63 ± 14.35 ^A^	58.13 ± 9.95 ^A^	40.00 ± 6.41 ^B^
Leptin (ng/mL)	162.3 ± 32.6 ^B^	986.3 ± 413.8 ^A^	429 ± 69.7 ^B^	303.2 ± 84.2 ^B^
Bili-total (mg/dL)	0.08 ± 0.01 ^A^	0.07 ± 0.01 ^A^	0.05 ± 0.01 ^B^	0.07 ± 0.02 ^A^
BUN (mg/dL)	15.33 ± 1.25 ^A^	9.55 ± 0.91 ^C^	12.80 ± 1.05 ^B^	10.96 ± 2.11 ^C^
Creatinine (mg/dL)	0.35 ± 0.05 ^AB^	0.38 ± 0.05 ^A^	0.31 ± 0.04 ^B^	0.38 ± 0.05 ^A^

Bili-total: total bilirubin; BUN: blood urea nitrogen. Control: normal diet; HFD: high-fat diet (60 kcal % fat); HFD + Pio: HFD (60 kcal % fat) + pioglitazone (30 mg/kg body weight); HFD + VA: HFD (60 kcal % fat) + vanillic acid (30 mg/kg body weight); (^A–C^) indicate statistically significant differences *p* < 0.05. Data are presented as mean ± SD (6 rats in each group).

### 3.5. Effect of Vanillic Acid on Hepatic Insulin Signaling, Inflammation and NEFA Formation in HFD-Fed Rats

**Figure 2:**
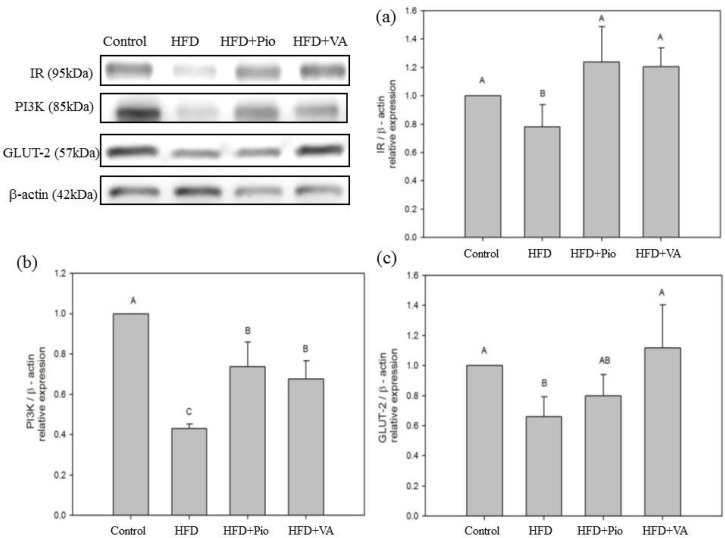
Hepatic (**a**) insulin receptor (IR); (**b**) phosphoinositide 3-kinase (PI3K); and (**c**) glucose transporter 2 (GLUT-2) proteins expression in rat fed high-fat diet for 16 weeks and orally administered vanillic acid during the last 4 weeks. Control: normal diet; HFD: high-fat diet (60 kcal % fat); HFD + Pio: HFD (60 kcal % fat) + pioglitazone (30 mg/kg body weight); HFD + VA: HFD (60 kcal % fat) + vanillic acid (30 mg/kg body weight); (A–C) indicate statistically significant differences *p* < 0.05. Data are presented as mean ± SD (3 rats in each group).

Insulin is a hormone with multiple effects. Binding of insulin to the α-subunit of the insulin receptor molecule induces rapid auto-phosphorylation of the β-subunit, which leads to an increase of its tyrosine kinase activity [43]. Tyrosine phosphorylation of insulin receptor proteins induces the cytoplasmic binding activity of insulin receptor substrate-1 (IRS-1) to insulin receptor. IRS-1 plays a pivotal role in transmitting signals from insulin receptors to intracellular PI3K/Akt pathway, which eventually results in the second intracellular step of insulin action, targeting tyrosine-phosphorylated insulin receptor β and IRS-1 [29,43,44,45]. IR is a condition in which defects in the action of insulin are such that normal levels of insulin do not operate as the signal for glucose uptake [43]. After a 4-week administration of VA, a down-regulation of hepatic insulin signaling-related proteins, such as IR, PI3K, and GLUT-2, was found in HFD group rats as compared with the control group (Figure 2). These results suggested that VA normalizes hepatic insulin signaling and alleviates hepatic insulin resistance in the liver of HFD-fed rats.

**Figure 3:**
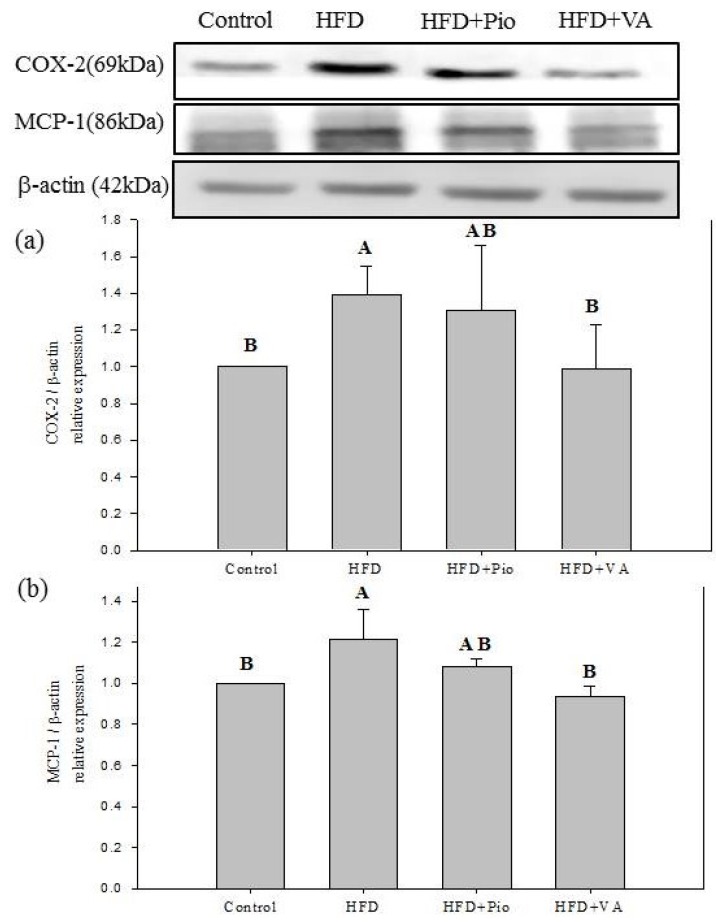
Hepatic (**a**) cyclooxygenase 2 (COX-2) and (**b**) monocyte chemoattractant protein-1 (MCP-1) protein expression in rats fed high-fat diet for 16 weeks and orally administered with vanillic acid during the last 4 weeks. Control: normal diet; HFD: high-fat diet (60 kcal % fat); HFD + Pio: HFD (60 kcal % fat) + pioglitazone (30 mg/kg body weight); HFD + VA: HFD (60 kcal % fat) + vanillic acid (30 mg/kg body weight); (A–C) indicate statistically significant differences *p* < 0.05. Data are presented as mean ± SD (3 rats in each group).

The chronic inflammation increases the development of obesity-related insulin resistance [5]. COX-2, an enzyme involved in inflammatory responses, is barely detectable in normal conditions and rapidly up-regulated in the inflammation conditions [28]. MCP-1, an inflammatory cytokine, is up-regulated in the conditions of hyperlipidemia and inflammation [46]. As shown in Figure 3, HFD up-regulated the hepatic COX-2 and MCP-1 protein expressions as compared with the control group. In contrast, the expressions of COX-2 and MCP-1 protein were down-regulated (*p* < 0.05) after a 4-week VA treatment in HFD-fed rats. Diet-induced obesity is a type of inflammatory response, with up-regulated COX-2 and MCP-1 protein expressions observed in the internal tissues of obese rats [47,48].

**Figure 4:**
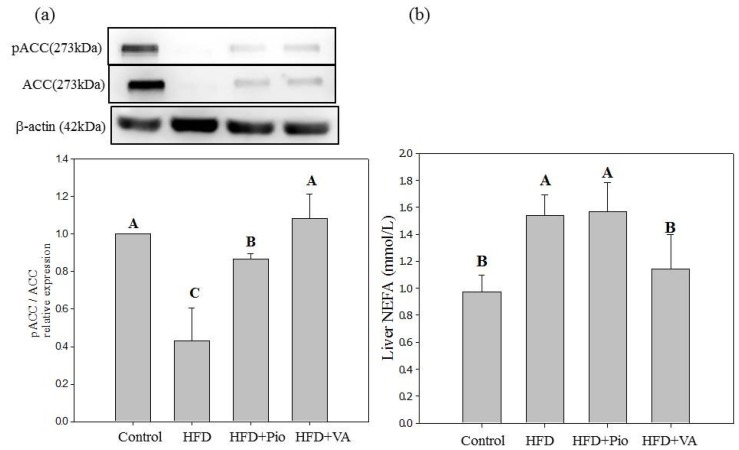
(**a**) Hepatic phosphorylated acetyl-CoA carboxylase/acetyl-CoA carboxylase (pACC/ACC) protein expression; and (**b**) non-esterified free fatty acid (NEFA) in rats fed high-fat diet for 16 weeks and orally administered with vanillic acid during the last 4 weeks. Control: normal diet; HFD: high-fat diet (60 kcal % fat); HFD + Pio: HFD (60 kcal % fat) + pioglitazone (30 mg/kg body weight); HFD + VA: HFD (60 kcal % fat) + vanillic acid (30 mg/kg body weight); NEFA: non-esterified free fatty acid in supernatant of liver homogenate. The liver tissue was homogenized with PBS (1/4; *w/v* and centrifuged at 4 °C for 60 min to obtain the supernatant; (A–C) indicate statistically significant differences *p* < 0.05. Data are presented as mean ± SD (6 rats in each group).

HFD increases the lipid synthesis in liver and adipose tissues, induces chronic inflammatory responses thus leading to insulin resistance in mice [28,37]. Acetyl CoA carboxylase (ACC), an essential enzyme in fatty acid synthesis, is activated by dephosphorylation and transforms acetyl CoA into malonyl CoA through carboxylation. Enhancing phosphorylation of ACC protein may thus reduce adipose tissue formation and NEFA accumulation in peripheral tissue, including liver [49]. Excess NEFA from adiposity was reported to cause insulin resistance by inhibiting insulin signaling in T2DM [49]. In the present study, the phosphorylated ACC/ACC protein expression of liver significantly decreased, indicating the increment of hepatic lipogenesis in the HFD rats (Figure 4a). The expression of hepatic phosphorylated ACC/ACC protein expression was increased, and the hepatic NEFA level reduced in HFD rats treated with VA for 4 weeks (Figure 4b). The current study suggests that VA may reduce hepatic NEFA accumulation via promoting the phosphorylation of ACC protein expression in liver of HFD rats.

Previous studies indicated that high concentration of NEFA was associated with increased expression of MCP-1 or COX-2 [50,51]. Recent reports also suggest that chronic MCP-1 or COX-2-mediated inflammation in fat is crucial for obesity-linked insulin resistance [52,53]. Phenolic acids were previously reported to exert counteractive effect on inflammation, and prevent the processing of chronic diseases [18]. The results from this study elucidate that VA may reduce hepatic NEFA level, decrease hepatic inflammatory responses and subsequently alleviate insulin resistance in HFD rats.

## 4. Conclusions

**Figure 5:**
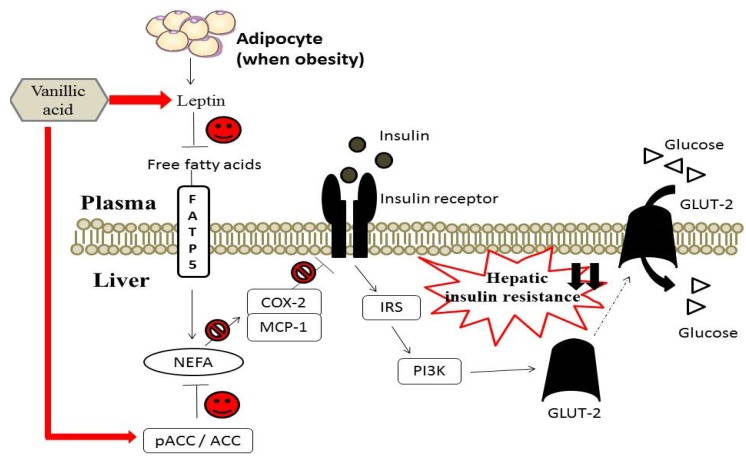
The postulated mechanisms underlying the effect of vanillic acid on hepatic insulin resistance by regulating insulin signaling and inflammation pathways in rats fed an HFD.

VA is a naturally occurring phenolic acid widely existing in various plant foods. The present study demonstrates that the protective effect of VA against hyperinsulinemia, hyperglycemia and hyperlipidemia is through decreasing hepatic NEFA accumulation, alleviating hepatic inflammation as well as hepatic insulin resistance in HFD-fed rats (Figure 5). Our findings support that VA exerts therapeutic effects and has the potential to be used in clinical medicine or as a dietary supplement for preventing the progression of DM.
